# Imaging of inner ear malformation in paediatric patients—a 10-year tertiary centre review

**DOI:** 10.1007/s00330-025-11663-5

**Published:** 2025-05-14

**Authors:** Yee Man Catherine Young, Ho Sang Leung, Wai Tsz Chang, Suet Mui Yu, Ki Wang, Ka Tak Wong, Chiu Wing Winnie Chu

**Affiliations:** 1 Department of Imaging and Interventional Radiology, Prince of Wales Hospital, The Chinese University of Hong Kong, Shatin, New Territories, Hong Kong; 2 Department of Otorhinolaryngology, Head and Neck Surgery, The Chinese University of Hong Kong, Shatin, New Territories, Hong Kong; 3 Institute of Human Communicative Research, The Chinese University of Hong Kong, Shatin, New Territories, Hong Kong

**Keywords:** Congenital, Hearing loss, Cochlear diseases

## Abstract

**Objective:**

Sennaroglu’s classification is the most accepted classification worldwide for inner ear and/or nerve malformation, although only limited data is available for the Asian population. This study aims to assess their relative frequency, summarise their radiological features, and determine their correlation with hearing impairment.

**Materials and methods:**

Retrospective analysis of patients with imaging-proven inner ear and/or nerve malformation in a single tertiary hospital in Hong Kong in 2014–2023. Clinical data were reviewed along with relevant imaging findings.

**Results:**

A total of 155 abnormal ears from 94 patients were included. All cases of Michel deformity (*n* = 4), rudimentary otocyst (*n* = 5), cochlear aplasia (*n* = 6) and common cavity (*n* = 3) show severe/profound hearing loss. Cochlear hypoplasia Type I (*n* = 2) and Type II (*n* = 6), and incomplete partition Type I (*n* = 5) also show severe/profound hearing loss; while cochlear hypoplasia Types III (*n* = 4) and Type IV (*n* = 8) and incomplete partition Type II (*n* = 28) show variable degrees of hearing deficit. Cochlear and vestibulocochlear nerve abnormalities were detected in 66% and 54% of cases, respectively. Generalised linear model analyses showed a correlation between the degree of hearing deficit with the presence of cochlear aperture atresia (OR = 2.562, *p* = 0.015), cochlear nerve atresia (OR = 2.599, *p* = 0.014), and vestibulocochlear nerve atresia (OR = 2.747, *p* = 0.064); and remained significant adjusting for the presence of cochlear abnormalities.

**Conclusion:**

A higher relative frequency of vestibulocochlear nerve and cochlear nerve abnormalities is observed in our study when compared with the existing literature. Cochlear nervous system abnormalities are most predictive of the degree of hearing impairment.

**Key Points:**

***Question*** *Accurate assessment of the inner ear/cochlear nerve is important for guiding treatment, but literature on their relative frequency and correlation with hearing impairment is lacking*.

***Findings*** *Cochlear system abnormalities are more frequent than reported in the literature. Cochlear aperture atresia, cochlear, or vestibulocochlear nerve aplasia are significant predictors of hearing impairment*.

***Clinical relevance*** *This study highlights the importance of assessment of the cochlear nervous system with the use of high-resolution MRI in the evaluation of paediatric sensorineural hearing loss*.

**Graphical Abstract:**

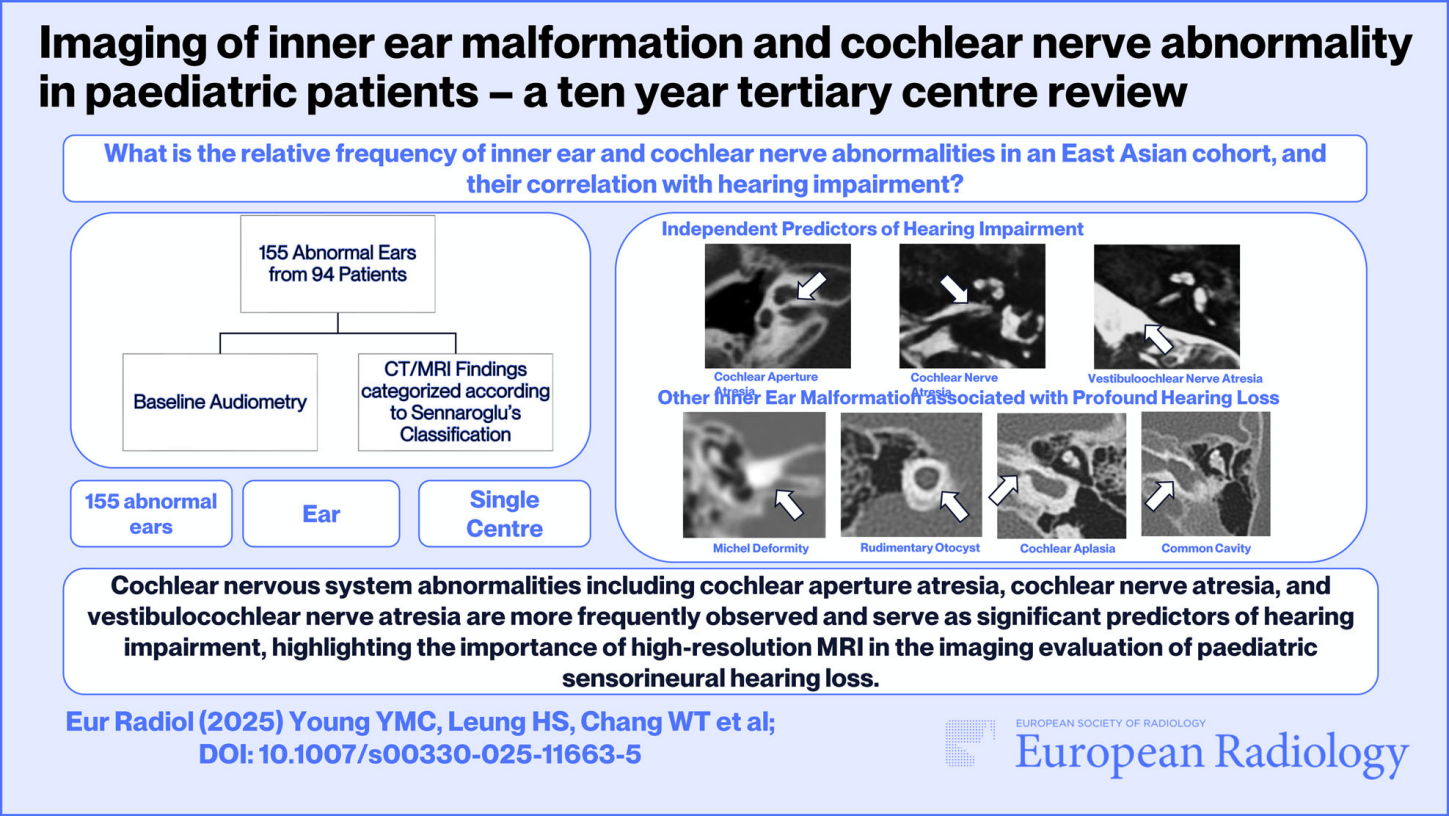

## Introduction

Congenital sensorineural hearing loss is a major cause of childhood disability, occurring in about 1.33–1.86 out of 1000 newborns [1, 2]. Amongst these affected children, 20% of them (i.e., 20–40 per 100,000 newborns) have underlying radiologically detectable inner ear malformations or associated nerve abnormality, while the remaining 80% of cases have cellular-level membranous abnormality that is not evident radiologically [3, 4]. Early detection and characterisation of the former group is of paramount importance for timely intervention and rehabilitation for these children [5–7], where hearing screening in Hong Kong is currently offered to all newborns via the universal newborn hearing screening programme or by Maternal & Child Health Centre, and, to all primary one and secondary two students by the Student Health Service Centre [8, 9].

Computed tomography (CT) and Magnetic resonance imaging (MRI) are important complementary imaging modalities in the evaluation of congenital hearing loss, looking into the osseous structures, anatomy of the vestibulocochlear apparatus and nerve structures [10–13]. Various classification systems have been proposed to classify the diverse findings of inner ear malformation or nerve abnormalities into subgroups due to the wide range of developmental arrest, which could occur at different stages of embryogenesis [14–16]. The latest Sennaroglu’s classification, revised in 2017 [17], is the most widely accepted classification, highlighting the imaging features of various malformations of each together with their audiological findings and management options. According to their recently published follow-up study [18], audiological and radiological findings are highly valuable in determining the appropriate treatment option between cochlear implantation and auditory brainstem implantation.

There have been limited studies worldwide regarding the prevalence of the various types of inner ear malformation and associated nerve abnormalities, especially in the Asian population. In this study, we aim to determine the relative frequency of various types of inner ear malformations and associated nerve abnormalities in our locality, describe their radiological features and determine their correlation with hearing impairment.

## Materials and methods

This is a retrospective study undertaken in a single tertiary hospital in Hong Kong after obtaining ethics approval from the Joint Chinese University of Hong Kong-New Territories East Cluster Clinical Research Ethics Committee (CREC No. 2024.031) dated 18 March 2024.

Medical records of all paediatric patients (0–18 years of age) who underwent CT petrous temporal bones, or MRI internal acoustic meatuses for hearing loss in the Department of Imaging and Interventional Radiology, Prince of Wales Hospital, Hong Kong, from 1st January 2014 to 31st December 2023 were reviewed. The cutoff age of 18 years is based on Sennaroglu’s landmark paper [15] and the age range of patients under paediatric ENT care in our locality. Available images of all patients were first reviewed by a single radiologist and cross-checked with the original report to exclude cases with normal imaging studies, sole malformation of the external or middle ear, or acquired causes of hearing loss. Cases with imaging-proven congenital inner ear or nerve malformation were included in the study.

For all included patients, their clinical data, including patient demographics, age and mode of presentation, as well as baseline audiometry, were obtained from the electronic patient record. In Hong Kong, the universal newborn hearing screening programme implemented in local birthing hospitals is adopting the two-stage automated auditory brainstem response screening protocol [9]. Neonates are screened in the maternity ward within the first 2–3 days of life, followed by a diagnostic auditory brainstem response test if screened positive in one or both ears. Audiological testing by an audiologist in a soundproof booth can test infants as young as 6 months behaviourally. VRA is used at the age of 6 months to 18 months, while play audiometry (PA) is applicable for ages 18 months to 5–6-years-old. Once a child is around 6–7-years-old, pure tone audiometry (PTA) tests will be applicable to them like an adult. The hearing threshold was calculated with the average of air conduction at 0.5 kHz, 1 kHz, 2 kHz, and 4 kHz of each tested ear. Degree of hearing impairment is classified into mild (26–40 dB), moderate (41–70 dB), severe (71–90 dB), and profound (91 dB or above), adopted from the American Speech-Language-Hearing Association (ASHA) [19]. Relevant imaging studies, if available, were independently reviewed by two radiologists (5 years and 8 years of experience, respectively) using the picture archiving and communication system, with discrepancies resolved by consensus. Imaging findings were categorised according to the latest Sennaroglu’s classification, revised in 2017 [17].

Descriptive statistics were used to describe the baseline demographics and malformation relative frequency. Correlation between the severity of hearing deficit with baseline demographics of age and sex, as well as the presence of each type of inner ear malformations, were performed using Spearman’s rho (for continuous variables), Mann–Whitney *U*-test (for binary variables) or Kruskal–Wallis test (for multinominal variables, additional pairwise comparisons with Bonferroni correction). Univariate ordinal logistic regression analysis was performed based on an ordinal scale of hearing deficit from the ASHA, with respective inner ear structural abnormalities. In addition, multivariate analyses were also performed to exclude confounding between inner ear and respective cochlear nerve malformations. Further sensitivity analyses were also performed with univariate and multivariate ordinal logistic regression, excluding cases with associated external or middle ear malformations. All statistical analysis was performed with SPSS version 26.0 (IBM Corp). Two-tailed *p* < 0.05 was considered statistically significant.

## Results

### Baseline demographics and audiometry

A total of 500 patients were retrieved from clinical records. After exclusion of cases performed for acquired conditions (82 patients) and patients without radiologically detected congenital anomaly other than inner ear malformations (324 patients, such as external auditory stenosis or ossicular chain dysplasia), a total of 94 patients with 155 abnormal ears were included in the final analysis. They comprised 51 (54.2%) male patients and 43 (45.7%) female patients with a mean age of presentation of 1.8 years (range 0–10-years-old).

Majority (*n* = 68, 72.3%) of the patients were identified during screening for hearing loss—most of which were identified by newborn hearing screening (*n* = 56, 59.6%), while the rest were revealed after neonatal period (*n* = 12, 12.8%) in maternal and child health centre (*n* = 1, 1%), child assessment centre (*n* = 6, 6.4%) or under student health service (*n* = 5, 5%). The remaining patients (*n* = 26, 27.7%) presented with symptomatic hearing loss.

Auditory brainstem response was the mode of hearing testing for the majority of the patients (*n* = 77, 81.9%) as most patients presented during newborn hearing screening. PTA was the second most common mode of audiology testing in our cohort (*n* = 10, 10.6%), followed by PA (*n* = 6, 6.4%) and VRA (*n* = 1, 1.1%).

**Fig. 1:**
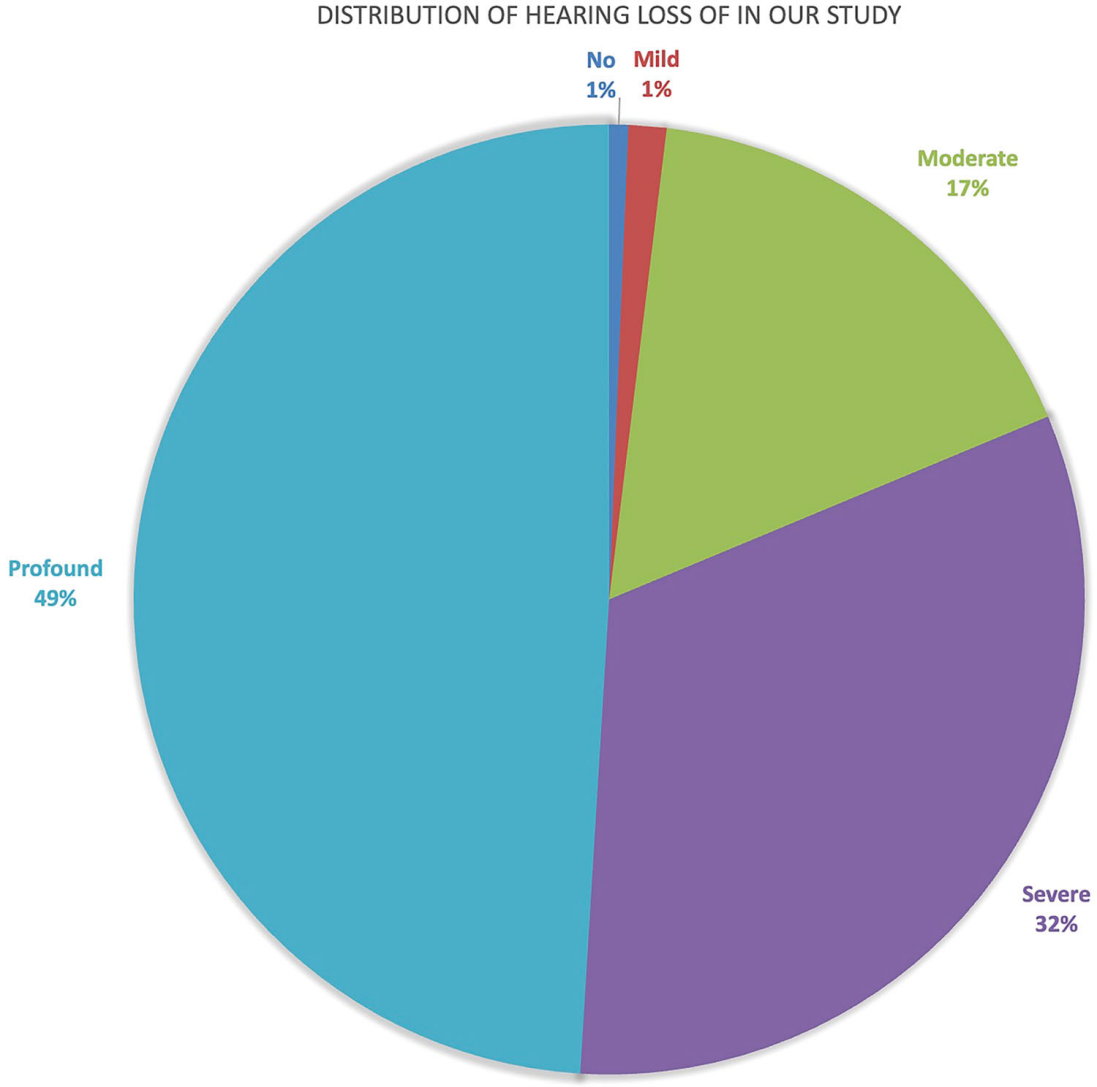
Pie chart showing the distribution of the degree of hearing impairment in our study

Almost half of the cases (*n* = 76, 49%) had a profound degree of hearing loss, with an additional 32.3% (*n* = 50) having a severe degree of hearing loss. Twenty-six cases (16.8%) had a moderate degree of hearing loss, while a mild degree of hearing loss was detected in two cases (1.3%). One case had no significant hearing loss (0.6%) where the patient had bilateral inner ear malformation, and the asymptomatic side was detected incidentally when imaging for bilateral ears was performed for the investigation of the contralateral side with a moderate degree of hearing loss (Fig. 1).

### Radiological assessment

CT temporal bones were available for review in 71.6% of cases (61/94 patients and 111/155 cases) while MRI internal acoustic meatuses were available for review in 90.3% of cases (86/94 patients and 140/155 cases). Both CT and MRI were performed for 61.9% of cases (52 patients and 96 cases). For MRI studies, 76.7% (66/86) of patients underwent imaging in a 3-T scanner, with the remainder scanned in the 1.5-T scanner. Out of the eight patients with no MRI available, seven of them were considered not eligible for cochlear implant, in view of satisfactory performance on conservative management or hearing aid (five patients) or with a significant conductive component (two patients). The remaining patient had an MRI performed in an outside institution, with images unavailable.

**Fig. 2:**
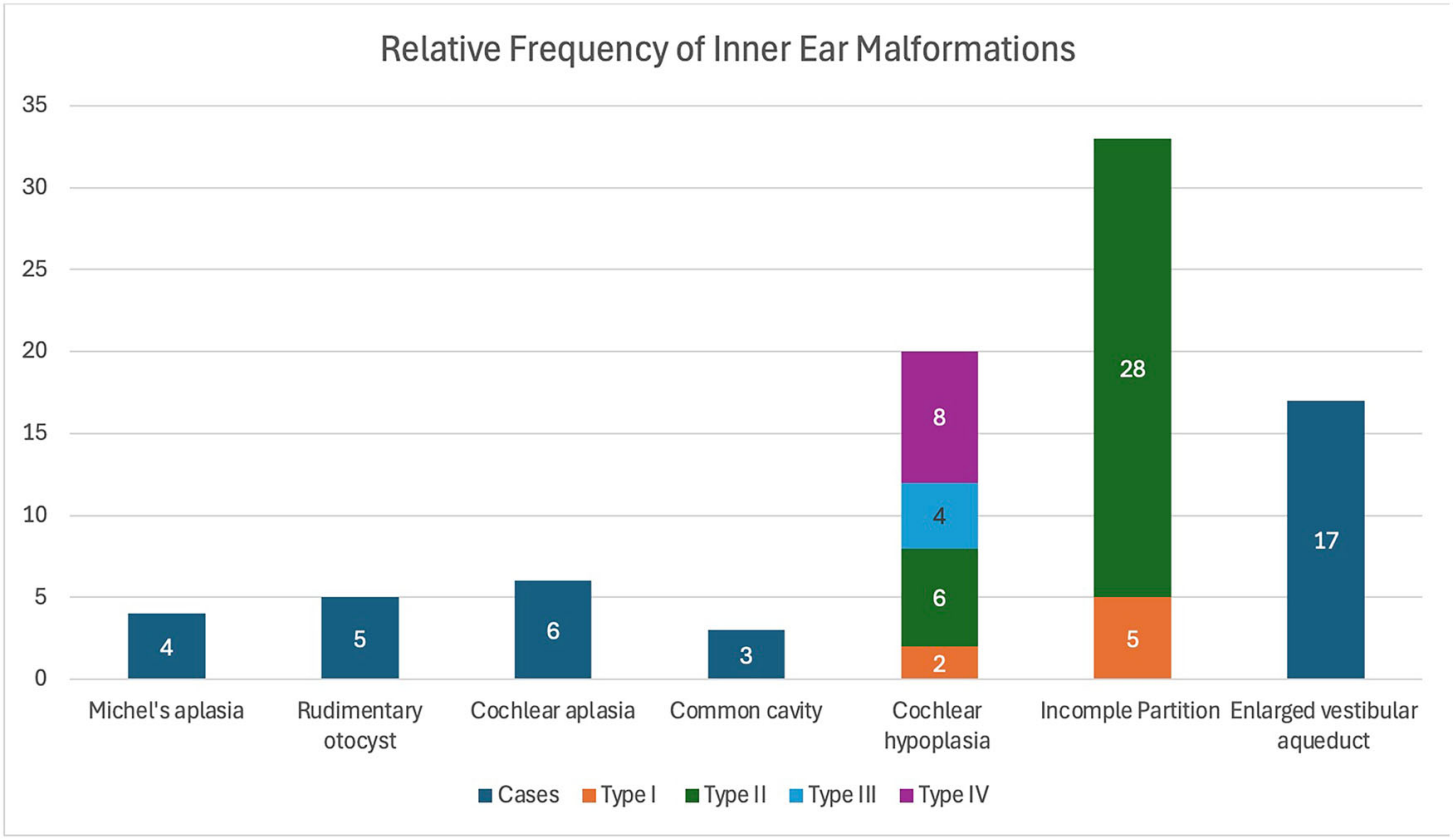
Bar chart showing the relative frequency of inner ear malformations according to Sennaroglu’s classification

### Imaging findings of inner ear malformations (Fig. 2)

Michel deformity is termed when the cochlea, vestibule and semicircular canal are completely absent within the osseous labyrinth [17, 20] and is the most severe deformity of all inner ear malformations [3, 20] (Supplementary Fig. 1A). This was seen in four cases in our cohort (2.6%), all of which had profound hearing loss.

Rudimentary otocyst is defined when there is only a tiny millimetric cystic structure within the osseous labyrinth with the absence of the normal internal auditory canal [3, 17, 20] (Supplementary Fig. 1B). This was detected in five cases in our cohort (3.2%), with a severe to profound degree of hearing loss.

Cochlear aplasia, which refers to the complete absence of the cochlea with the vestibule and semicircular canal being in their normal location [3, 17, 20] (Supplementary Fig. 1C), is demonstrated in six cases in our cohort (3.9%) presenting with a severe to profound degree of hearing loss.

Common cavity syndrome is described as a single cystic chamber consisting of the fused cochlear and vestibule with a lack of normal differentiation [3, 17, 20] (Supplementary Fig. 1D). The internal auditory canal is present, contrasting it from the rudimentary otocyst, entering through the centre of the cavity [3, 17, 20]. Three cases (1.9%) of common cavities were identified in our cohort with severe to profound hearing loss.

Cochlear hypoplasia, which is characterised by a small-sized cochlea but maintains the normal differentiation with the adjacent vestibule [17, 20], is detected in 20 cases in our cohort. It is subdivided into four types depending on the degree of internal architectural malformation [17, 20]. Cochlear hypoplasia Type I (Supplementary Fig. 2A) is the most severe form, where there is only a bud-like cochlea with non-identifiable internal architecture [17, 20], and is seen in two cases (1.3%) in our cohort with profound hearing loss. In cochlear hypoplasia Type II (Supplementary Fig. 2B), the normal external appearance of the cochlea is maintained while the modiolus and internal septa are malformed [17, 20]. This is seen in six cases (3.9%) in our cohort with severe to profound hearing loss. Cochlear hypoplasia Types III and IV are generally considered less severe, with Type III (four cases) demonstrating less than two cochlear turns accompanied with shortened modiolus (Supplementary Fig. 2C) while Type IV (eight cases) shows a preserved basal turn but hypoplasia involving the middle and apical turns (Supplementary Fig. 2D) [17, 20]. Three cases of cochlear hypoplasia Type III and three cases of cochlear hypoplasia Type IV presented with severe to profound hearing loss, with the presence of concomitant aplastic/hypoplastic cochlear nerves and/or apertures, while the remaining patients showed no other hearing abnormalities and had only up to moderate hearing loss.

Incomplete partition, on the other hand, is characterised by variable degrees of internal architectural deficits but maintains a normally sized cochlea with normal differentiation from the vestibule [17, 20]. It is further subdivided into three types depending on the degree of internal architectural malformation [17, 20]. Incomplete partition Type I refers to the cystic appearances of the cochlea and is commonly associated with dilated vestibule (Supplementary Fig. 3A, B) [17, 20]. This is seen in five cases (3.2%) in our cohort, all with a severe to profound degree of hearing loss. Incomplete partition Type II shows preserved basal turn but with cystic coalescence of the middle and apical turn (Supplementary Fig. 3C, D) [17, 20]. This is detected in 28 cases (18.1%) in our cohort associated with variable degrees of hearing loss. Type III incomplete partition, where there is preserved interscalar septa but aplastic modiolus [17, 20] is not seen in our cohort.

Enlarged vestibular aqueduct, which is defined by abnormal dilatation (> 1.5 mm) of the vestibular aqueduct measured at its mid portions [3, 17, 20] (Supplementary Fig. 4), is seen in six cases (3.87%) as an isolated abnormality with additional 37 cases associated with other anomalies (23.9%) in our cohort. It is associated with a moderate to severe degree of hearing loss.

Cochlear aperture refers to the opening at the base of the cochlea where the cochlear nerve enters the cochlea. 41 cases (26.5%) in our cohort shows stenosis of the cochlear aperture (Supplementary Fig. 5A) while 40 cases (25.8%) show complete atresia of the cochlear aperture, where it is replaced by the bone (Supplementary Fig. 5B). It is associated with moderate to profound degree of hearing loss.

### Imaging findings of cochlear nerve abnormalities

**Fig. 3:**
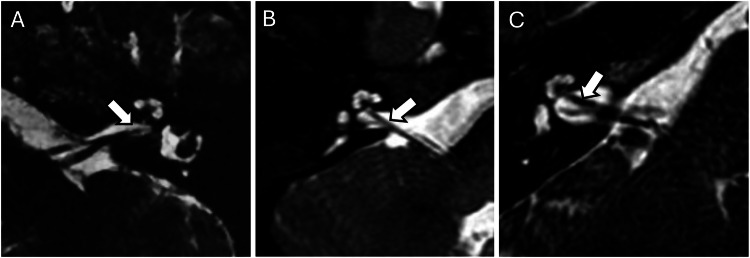
MRI internal acoustic meatuses illustrating cochlear nerve abnormalities. **A** Cochlear nerve aplasia with absence of the cochlear nerve entering the cochlear aperture (white arrow). **B** Cochlear nerve hypoplasia with appreciation of the cochlear nerve but with reduced calibre (white arrow). **C** Normal cochlear nerve (white arrow) as comparison

**Fig. 4:**
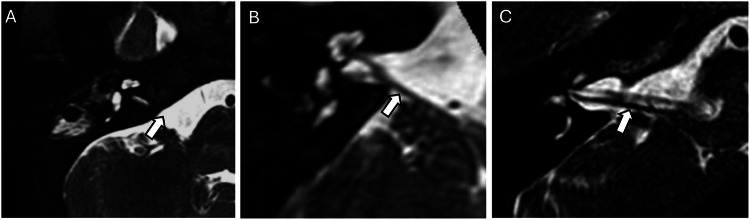
MRI internal acoustic meatuses illustrating vestibulocochlear nerve abnormalities. **A** Vestibulocochlear nerve aplasia with absence of the vestibulocochlear nerve entering the internal acoustic meatus (white arrow). **B** Vestibulocochlear nerve hypoplasia with appreciation of the vestibulocochlear nerve but with reduced calibre (white arrow). **C** Normal vestibulocochlear nerve (white arrow) as comparison

Cochlear nerve and vestibulocochlear nerve abnormalities are equally important in the assessment of congenital hearing loss. Cochlear nerve hypoplasia was detected in 38 cases (27.1%) while aplasia of the cochlear nerve was detected in 54 Cases (38.6%) (Fig. 3). Hypoplasia of the vestibulocochlear nerve, which is the more proximal portions of the cochlear nerve before it trifurcates with the vestibular nerve, was detected in 59 Cases (42.1%) while aplasia of the vestibulocochlear nerve was detected in 17 Cases (12.1%) (Fig. 4).

### Correlation with the severity of hearing loss at presentation

The age of presentation shows a weak but significant correlation with the degree of hearing deficit (Spearman’s rho = 0.192, *p* = 0.018), while the degree of hearing deficit does not differ across sex (*p* = 0.394 by Mann–Whitney *U*-test). There have been significant differences in the severity of hearing loss between different modes of presentation (*p* = 0.013 by Kruskal–Wallis test), with pairwise comparison showing more severe hearing loss in patients presented during neonatal newborn screening than those with hearing impairment later in development (adjusted *p* = 0.011).

The presence of any cochlear abnormality showed no statistically significant difference with a degree of hearing deficit on baseline audiometry (*p* = 0.732 by Mann–Whitney *U*-test), while the individual types of inner ear malformations, except for cochlear aperture abnormality, also show no significant correlation (Table 1). Univariate and multivariate ordinal logistic regression analyses demonstrated significant correlation between degree of hearing deficit with the presence of cochlear aperture atresia (OR = 2.562, *p* = 0.015), cochlear nerve atresia (OR = 2.599, *p* = 0.014), and vestibulocochlear nerve atresia (OR = 2.747, *p* = 0.064) (Table 2); these results remain significant in multivariate regression adjusting for the presence of cochlear abnormalities. These results also remain similar upon additional sensitivity analyses, with repeated regression analyses after exclusion of cases with concomitant middle or external ear malformations (Table 3).

**Table 1:** Incidence of inner ear abnormalities in our cohort according to Sennaroglu’s classification and the correlation with hearing deficit

	Cases	Correlation with degree of hearing deficit (*p* value)
Michel’s aplasia	4 (2.6%)	0.089^a^
Rudimentary otocyst	5 (3.2%)	0.182^a^
Cochlear aplasia	6 (3.9%)	0.325^a^
Common cavity	3 (1.9%)	0.549^a^
Cochlear hypoplasia	20 (12.9%)	0.286^a^
Type I	2 (1.3%)	
Type II	6 (3.9%)	
Type III	4 (2.6%)	
Type IV	8 (5.2%)	
Incomplete partition	33 (31.3%)	0.336^b^
Type I	5 (3.2%)	
Type II	28 (18.1%)	
Enlarged vestibular aqueduct	17 (11.0%)	
Isolated abnormality	6 (3.9%)	
Associated with other anomalies	11 (7.3%)	
Cochlear aperture abnormality		**
Narrowing	40 (25.8%)	
Absence	41 (26.5%)	
Cochlear nerve abnormality		**
Hypoplasia	38 (27.1%)	
Aplasia	54 (38.5%)	
Vestibulocochlear nerve abnormality		**
Hypoplasia	59 (42.1%)	
Aplasia	17 (12.1%)	

Cases are represented in numbers (percentages)

^a^ By Mann–Whitney *U*-test

^b^ By Kruskal–Wallis test

^**^ Refer to Table 2

**Table 2:** Univariate and multivariate ordinal logistic regression of the presence of cochlear nerve developmental abnormalities with hearing deficit

Parameter	Univariate regression		Multivariate regression^c^	
	*p* value	OR (95% CI)	*p* value	OR (95% CI)
Presence of CA atresia^a^	*p* = 0.019	2.47 (1.16–5.27)	*p* = 0.020	2.46 (1.15–5.26)
Presence of CA hypoplasia^a^	*p* = 0.662	1.23 (0.60–2.51)	*p* = 0.568	1.25 (0.58–2.67)
Presence of CN atresia^b^	*p* = 0.014	2.60 (1.22–5.56)	*p* = 0.011	2.71 (1.26–5.83)
Presence of CN hypoplasia^b^	*p* = 0.779	1.12 (0.50–2.50)	*p* = 0.587	1.26 (0.54–2.94)
Presence of VCN atresia^b^	*p* = 0.064	2.75 (0.95–7.99)	*p* = 0.052	2.90 (0.99–8.51)
Presence of VCN hypoplasia^b^	*p* = 0.270	1.46 (0.74–2.88)	*p* = 0.163	1.66 (0.82–3.37)

*OR* odds ratio, *CA* cochlear aperture, *CN* cochlear nerve, *VCN* vestubulocochlear nerve, *NS* not significant

^a^ Include all 155 cases

^b^ Include 140 cases with MRI performed

^c^ Adjusted for the presence of any cochlear abnormalities

**Table 3:** Univariate and multivariate ordinal logistic regression of the presence of cochlear nerve developmental abnormalities with hearing deficit (after exclusion of cases with middle or external ear deficit)

Parameter	Univariate regression		Multivariate regression^c^	
	*p* value	OR (95% CI)	*p* value	OR (95% CI)
Presence of CA atresia^a^	*p* = 0.011	3.061	*p* = 0.010	3.085
		(1.298–7.215)		(1.307–7.278)
Presence of CA hypoplasia^a^	*p* = 0.421	1.389	*p* = 0.298	1.569
		(0.624–3.095)		(0.672–3.661)
Presence of CN atresia^b^	*p* = 0.011	2.900	*p* = 0.010	3.413
		(1.279–6.573)		(1.454–8.010)
Presence of CN hypoplasia^b^	*p* = 0.814	1.106	*p* = 0.426	1.447
		(0.478–2.562)		(0.582–3.597)
Presence of VCN atresia^b^	*p* = 0.083	2.761	*p* = 0.049	3.247
		(0.876–8.699)		(1.004–10.506)
Presence of VCN hypoplasia^b^	*p* = 0.153	1.696	*p* = 0.049	2.193
		(0.821–3.503)		(1.002–4.800)

*OR* odds ratio, *CA* cochlear aperture, *CN* cochlear nerve, *VCN* vestubulocochlear nerve

^a^ Include 140 cases with only inner ear malformations

^b^ Include 126 cases with only inner ear malformations and an MRI performed

^c^ Adjusted for the presence of any cochlear abnormalities

## Discussion

To the best of our knowledge, this is the first retrospective study looking into both CT and MRI findings of various types of paediatric inner ear malformation in an East Asian population.

Our current study has also shown a weakly positive, but statistically significant, correlation between the presenting age and hearing deficit upon presentation, while no significant correlation is seen with gender. We believe the former apparent correlation is likely due to selection bias from our tertiary centre, where a threshold for imaging and further management is often lower for younger patients, where a milder degree of hearing deficit would still impact speech and developmental outcomes.

Within our cohort, the distribution of inner ear malformations is similar to those reported in international literature. Incomplete partition, being one of the later occurring cochlear malformation in fifth to seventh week of gestation [21, 22], is the most commonly detected inner ear malformation in our study accounting to 31.3% of our whole cohort, and 33/71 (46.4%) patients amongst those with MRI-detected inner ear malformations; the latter of which consistent with the results from other studies worldwide (ranging from 41% to 84% of all malformations identified on MRI) [17, 21, 23]. Cochlear hypoplasia is the second most common inner ear malformation, rounding up to 13% in our cohort. This is again in line with previous literature with a quoted figure between 9% and 12% by Sennaroğlu [16], Sun et al [23], Tahir et al [24], and Tanon-Anoh et al [25]. Michel deformity, rudimentary otocyst, cochlear aplasia, and common cavity syndrome are seen as isolated cases in our cohort (< 5%), with similar prevalence as previous literature, including ranging from 2% to 5% [16, 23–25]. Enlarged vestibular aqueduct is rare as an isolated abnormality in our cohort (3.87%), but rounds up to a significant proportion (27.7%) when associated with other abnormalities. This is significantly lower than other published literature with quoted figures of 33–40% [23, 26] as an isolated abnormality and 56–62% [26] when associated with other abnormalities [21]. The underlying cause for this is uncertain, but its being more commonly associated with other additional inner ear anomalies is in line with other studies [3].

Cochlear aperture abnormality was shown in over half of the cases (52% of cases using a cut-off of 1.4 mm) in our cohort which is like other studies by Orzan et al (70% using a cut-off of 1.2 mm) [27] and Lim et al (61% using a cut-off of 1.4 mm) [28]. Cochlear nerve abnormalities and vestibulocochlear abnormalities were more commonly detected in our population as compared to the other studies. Cochlear nerve hypoplasia or aplasia was detected in 66% of our cohort but was only present in 50% in the study by Lipshitz et al [29], 45% by Tahir et al [24], 36% by Orzan et al [27], as well as 18% by Tanon-Anoh et al [25] and McClay et al [30]. Vestibulocochlear nerve abnormality, either hypoplasia or aplasia, was detected in almost half of our cohort (54%) while it was only detected in 30% in Agarwal et al [21]. The exact reason for this observation remains uncertain, but we postulate this could be attributed to higher resolution of the 3-T MR scanner in our institute where most (over 75%) of our studies were performed which allows us to more precisely characterise and assess the status of the vestibulocochlear and cochlear nerve while previous studies by Orzan et al [27] and McClay et al [30] were performed on 1.5-T scanner. Further larger-scale research encompassing diverse ethnic groups, including the Caucasian population, would be beneficial to validate the findings.

Our study also showed that the abnormalities of the cochlear nervous system, including cochlear aperture, cochlear nerve and/or vestibulocochlear nerve, correlate with the degree of hearing loss upon presentation. This is in line with the results previously published by Wilkins regarding cochlear aperture stenosis [31], and Vos on cochlear nerve deficiency, including both aplasia and hypoplasia [32]. Furthermore, in our current study, we showed the absence of cochlear aperture and atresia of the cochlear or vestibulocochlear nerves showed a stronger, significant correlation compared to hypoplasia of these structures, concurring with the concept that residual fibre density would correlate with hearing function. These results remain robust after adjusting for the presence of any cochlear malformation and after additional analyses. On the other hand, the current results were unable to demonstrate a significant correlation with hearing deficit with each of the other inner ear malformations, which is likely due to the rarity of these cases, resulting in an inadequacy of statistical power. Nevertheless, when results were analysed in a broader perspective, the presence of any inner ear malformation also shows no significant correlation with hearing loss severity, further validating the independent predictive role of the cochlear nervous system on hearing.

The above findings are also supported from the perspective of embryological pathogenesis, as various types of paediatric inner ear malformation arise due to developmental arrest at different stages. Otic placode develops as early as three weeks of gestation with subsequent differentiation of cochlear, vestibule and semicircular canals [22, 32, 33]. Insults at earlier stages of development in the third to fifth week result in more severe deformities such as Michel deformity, rudimentary otocyst, cochlear aplasia, common cavity syndrome [3, 14, 22, 34, 35]. This is described to be associated with profound degrees of hearing loss by Sennaroglu’s classification [17], which is consistent with our results despite being a small cohort. Development and differentiation of the cochlea later occur in the sixth to eighth week [14, 22, 35] with a variable degree of hearing loss depending on the timing of insult and severity of malformation, as observed in our cohort, again consistent with Sennaroglu’s classification [17]. Vestibular aqueduct develops alongside the cochlea, possibly due to a common developmental precursor, with progressive narrowing during the fifth to eighth week of gestation [14, 36]. It is associated with variable degrees of hearing loss and is frequently associated with other inner ear malformations [3] as seen in our cohort. The development of the cochlear aperture and cochlear nerve is hand in hand [33]. The formation of the vestibulocochlear nerve begins at three weeks of gestation, followed by the formation of the internal auditory canal at nine weeks of gestation [3, 37, 38]. Most of the cases of cochlear aperture atresia or hypoplasia is therefore associated with cochlear nerve abnormality [33, 38], which is best assessed on MRI as in our study, where we demonstrated that only atresia (but not hypoplasia) of the cochlear or vestibulocochlear nerves are associated with significantly worse baseline hearing outcome.

This study is the first on radiological manifestations of inner ear malformations in an East Asian population over 10 years. Following a standardised classification of inner ear malformations, we can accurately quantify the relative frequency and distribution of this group of heterogeneous conditions. Our relative frequency and distribution of hearing impairment are largely similar to the other cohorts reported, apart from the higher relative frequency of cochlear or vestibulocochlear nerve atresia, which can be related to the advances in MR technology and resolution, resulting in better CN VII, VIII nerve recognition.

A few limitations of this study remain. To start with, despite a comprehensive review period spanning over 10 years, our single-centre research only yielded a moderate sample size with unimaged cases of mild degree of hearing loss also inherently precluded introducing potential bias. Future larger-scale research studies involving other centres in our locality and the greater China would help validate our local data. In addition, the retrospective nature of our study only allowed us to focus on a review of clinical charts and previous imaging records. Being a tertiary centre with some patients receiving imaging before referral, the imaging protocol is also inconsistent. The presence of an insufficient masking phenomenon (i.e., audiometric assessment of a severely abnormal ear being compensated by the other normal/ mildly affected ear) may also create bias for audiometry findings. Last, the methodology of exclusion with single read was also less robust when compared to the scrutiny of imaging findings with double read, where potentially additional positive cases of inner ear malformation may be excluded.

Future large-scale studies in our region, encompassing the greater China and other ethnicities, can validate our local data regarding relative frequency and the relationship between the development of the cochlear nerve and the severity of hearing loss. Additionally, the relationship between genetics and various types of inner ear malformations, as well as the efficacy of different treatment approaches, are areas that warrant further research in the advancement of the field of paediatric and otorhinolaryngology. The increasing importance of genetics in predicting congenital sensorineural hearing loss has been highlighted in recent research [39, 40], while the translation of treatment into developmental outcomes still warrants further study [41, 42].

## Conclusion

The current study summarised the spectrum and complexity of paediatric inner ear malformations on imaging of an East-Asian population, with the higher relative frequency of cochlear and vestibulocochlear nerve abnormalities observed when compared to prior studies, which could be attributed to the higher resolution of 3-T scanners employed. A significant correlation was also identified between the abnormal development of the cochlear nervous system and the degree of hearing deficit, independent of any inner ear malformations. Future studies involving a broader and more diverse population would be beneficial in validating the above findings. Further research on the genetic basis of various inner ear malformations and the efficacy of different treatment modalities can also contribute to advancements in this field.

## Supplementary information

ELECTRONIC SUPPLEMENTARY MATERIAL
