# Supplementary material for: Imaging of inner ear malformation in paediatric patients—a 10-year tertiary centre review
Source: Eur Radiol. 2025 May 14;35(11):6735–44. doi: 10.1007/s00330-025-11663-5 (PMC12559043; doi:10.1007/s00330-025-11663-5)

Imaging of Inner Ear Malformation in Paediatric Patients – A Ten Year Tertiary Centre Review

ELECTRONIC SUPPLEMENTARY MATERIAL

Eur Radiol (2025) Young YMC, Leung HS, Chang WT, et al.

Supplementary Figure 1. CT Petrous Temporal Bones illustrating various cochlear anomalies.

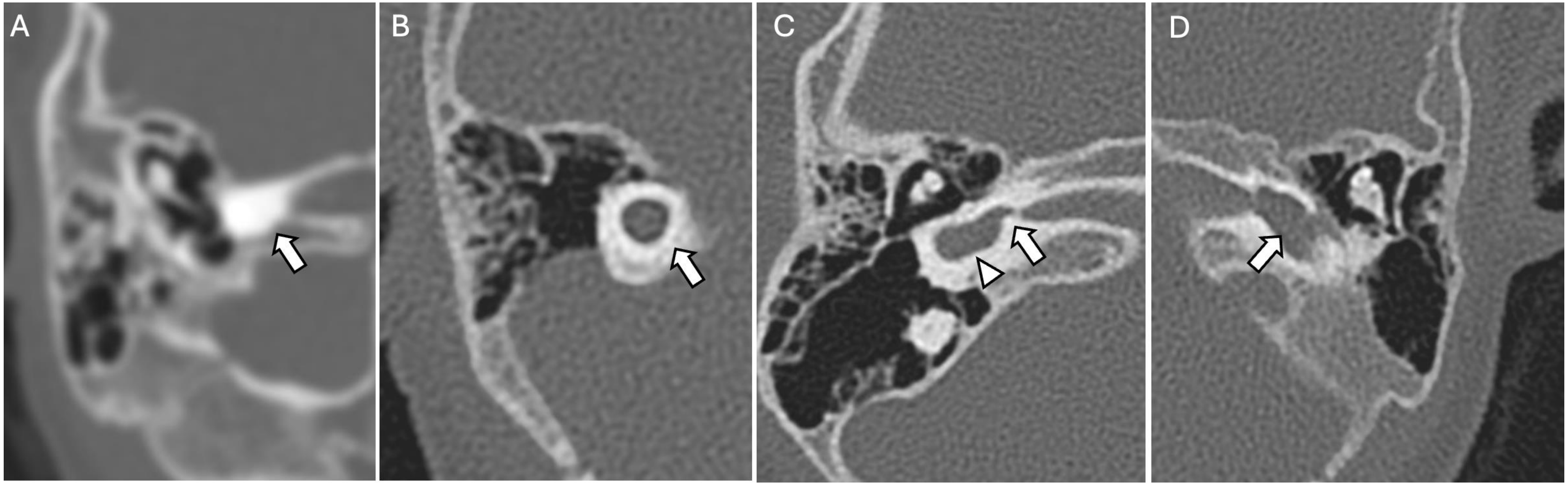

(A) Michel deformity characterized by abnormal sclerosis of the petrous temporal bone with lack of development of membranous labyrinth (white arrow). (B) Rudimentary otocyst characterized by small ovoid cyst representing rudimentary membranous labyrinth with lack of normal internal auditory canal (white arrow). (C) Cochlear aplasia demonstrated by complete absence of cochlea (white arrow) with cystic dilatation of vestibule (white arrowhead). (D) Common cavity refers to a common cystic structure comprising of the undifferentiated cochlear and vestibule (white arrow).

Supplementary Figure 2. CT Petrous Temporal Bones and MRI Internal Acoustic Meatus illustrating various types of cochlear hypoplasia.

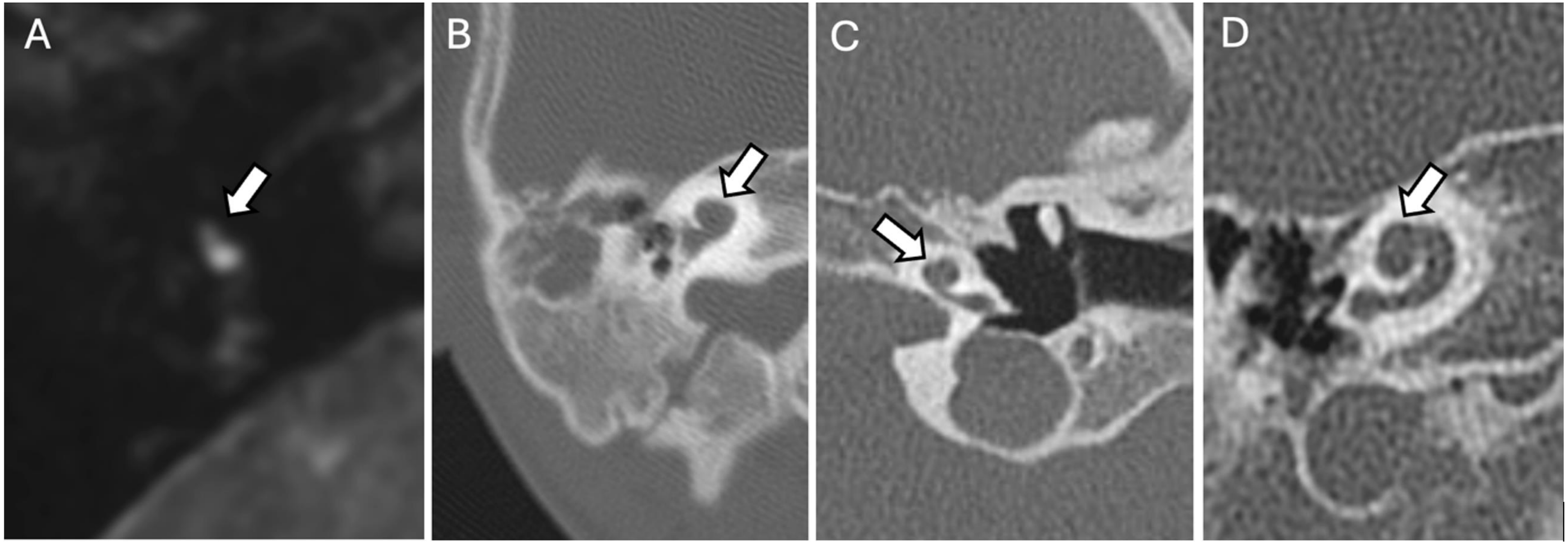

(A) Cochlear hypoplasia type I represented by a bud-like appearances of the cochlea (white arrow). (B) Cochlear hypoplasia type II shows preserved external architecture of the cochlea but loss of normal modiolus (white arrow). (C) Cochlear hypoplasia type III as illustrated by fewer than two turns of the cochlea (white arrow). (D) Cochlear hypoplasia type IV shows hypoplasia involving the middle and apical turns but with preserved basal turn (white arrow).

Supplementary Figure 3. Various types of incomplete partition in our cohort illustrated on CT Petrous Temporal Bones and MRI Internal Acoustic Meatus.

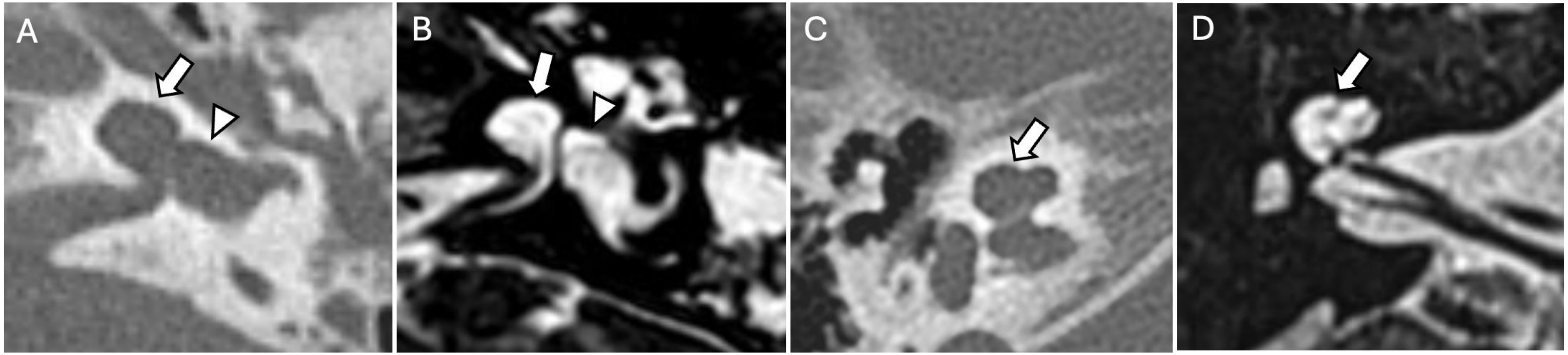

(A, B) Incomplete partition type I represented by cystic appearances of both the cochlea (white arrows) and vestibule (white arrowheads). (C, D) Incomplete partition type II shows preserved basal turn but with cystic coalescence of middle and apical turns (white solid arrows).

Supplementary Figure 4. Enlarged vestibular aqueducts illustrated on CT Petrous Temporal Bones (A) and MRI Internal Acoustic Meatus (B).

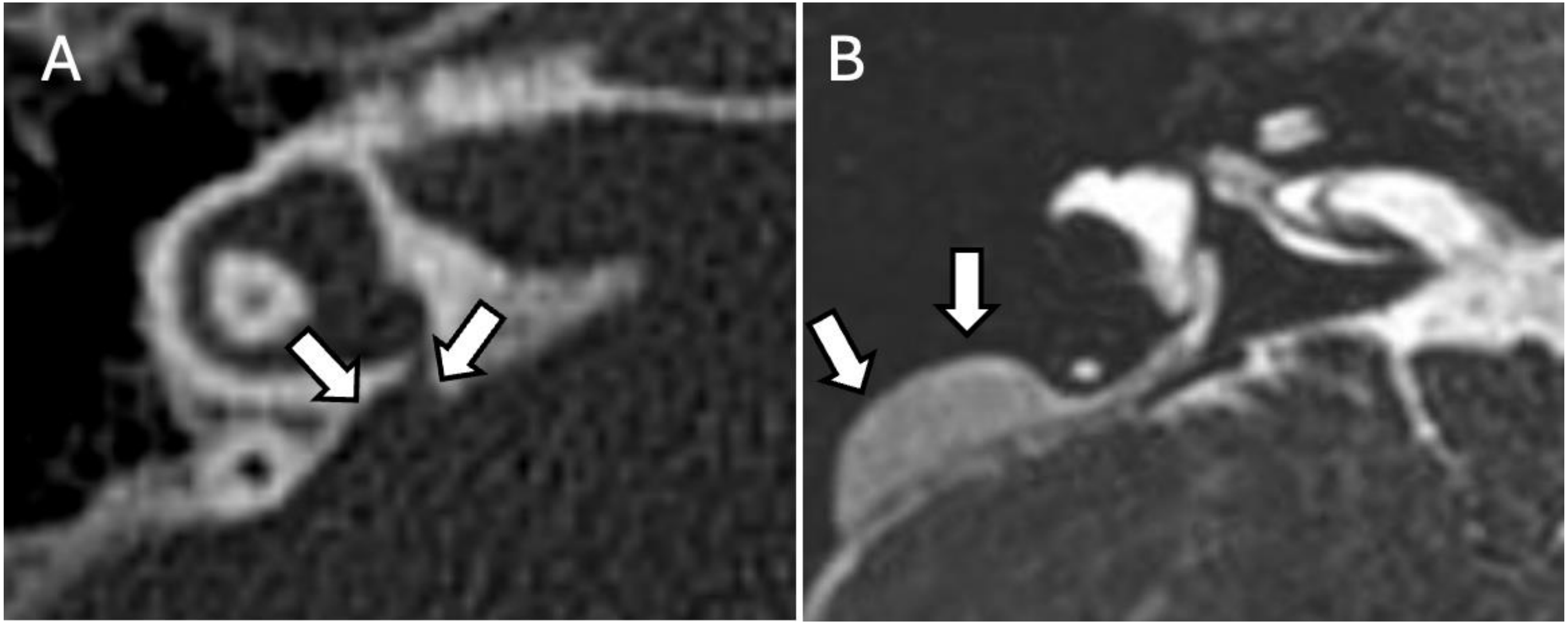

Supplementary Figure 5. CT Petrous Temporal Bones illustrating hypoplasia of the cochlear aperture (white arrow in A) and atresia of the cochlear aperture (white arrow in B).

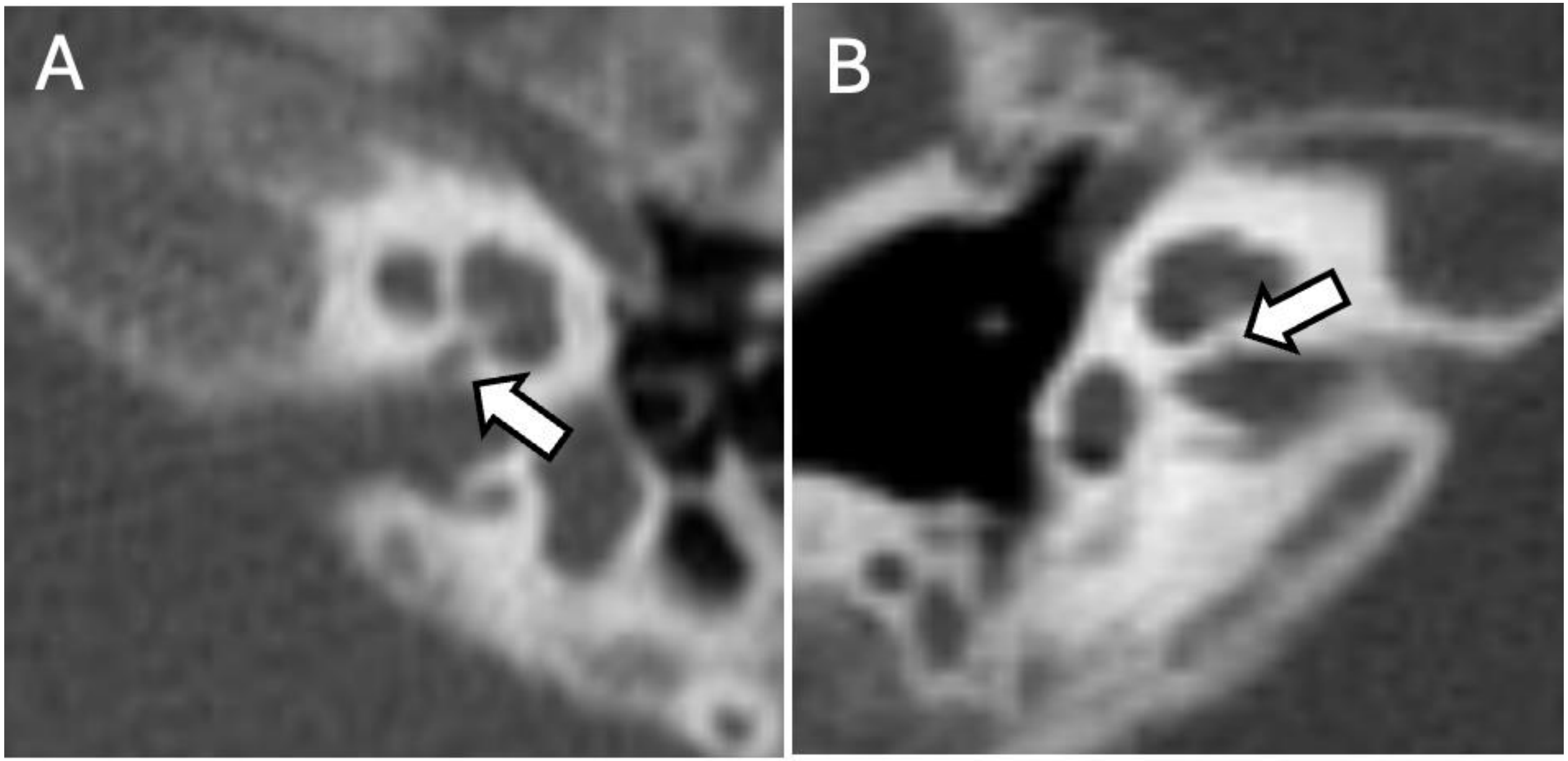

Supplement: Supplementary file 1 — ELECTRONIC SUPPLEMENTARY MATERIAL [file 330_2025_11663_MOESM1_ESM.pdf]
